# 48 week bone marker changes with Dolutegravir (DTG) plus Abacavir/Lamivudine (ABC/3TC) vs. Tenofovir/Emtricitabine/Efavirenz (EFV/TDF/FTC): the SINGLE trial

**DOI:** 10.1186/1471-2334-14-S2-P72

**Published:** 2014-05-23

**Authors:** Y Yazdanpanah, M-A Khuong-Josses, L Hocqueloux, G Pialoux, J Durant, B Wynne, C Granier, P Tebas, K Pappa, S Min

**Affiliations:** 1 Bichat Claude-Bernard Hospital - APHP, Paris, France

## Background

DTG is a once-daily integrase inhibitor that in combination with ABC/3TC, demonstrated superior efficacy with favorable tolerability over TDF/FTC/EFV (SINGLE study) at 48 weeks. EFV is associated with reduced Vitamin D levels through CYP450 enzyme induction; TDF has been associated with decreased bone mineral density (BMD). Antiretroviral treatment (ART) initiation is characterized by initial rapid decline in bone mineral density (BMD) that subsequently stabilizes. Bone turnover markers are increased during these BMD changes.

## Methods

In the double-blind SINGLE study (table 1) we measured markers of bone turnover (bone-specific alkaline phosphatase, C-termial telopeptide type 1 collagen, osteocalcin, and procollagen type 1 N-propeptide) & Vitamin D at baseline (BL) and at 48 weeks. ANCOVA analyses were adjusted for the following factors age, sex, HIV RNA, CD4+ cell count, BL biomarker level, BMI, smoking status, and Vitamin D supplementation.

**Table 1:** 

	DTG+ABC/3TC change from baseline* (N=414)	TDF/FTC/EFV change from baseline* (N=419)	Geometric Mean Ratio**	[95% CI for the ratio] p-value
Bone-specific alkaline phosphatase	15%	60%	0.720	[0.685-0.756] <0.001

C-terminal telopeptide for type 1 collagen	33%	68%	0.789	[0.747-0.832] <0.001

Osteocalcin	22%	48%	0.827	[0.779-0.878] <0.001

Procollagen type 1 N-propeptide	30%	66%	0.784	[0.749- 0.821] <0.001

Vitamin D (25-hydroxy-Vitamin D)	-7%	-10%	1.036	[0.970-1.107] P=0.292

*adjusted percent change from baseline=100 X adjusted geometric mean of (week 48/baseline)-100

**GMR= (DTG+ABC/3TC+100) / (TDF/FTC/EFV+100)

## Results

833 subjects were analyzed: 84% males; 68% whites. Mean BL VL: 4.7 log10 c/mL; 32% VL>100,000 c/mL and mean BL CD4 cell count = 350 cells/mm3. Bone markers increased in both groups but the increases were significantly greater in subjects treated with TDF/FTC/EFV. Vitamin D levels decreased in both treatment groups; the differences were not significant between groups.

## Conclusions

After 48 weeks, significantly greater changes from baseline were observed for all bone markers in subjects receiving TDF/FTC/EFV, indicating more active bone turnover when compared to changes seen in subjects receiving DTG + ABC/3TC. These differences may correlate with known TDF-associated changes in BMD over time and further study of the potential advantages of a DTG+ABC/3TC regimen appear warranted.

